# Styes

**Published:** 1907-05

**Authors:** 


### STYES.

Styes occur at all ages, but they are more common in children and young adults, and often appear in crops. As a rule, the patient is out of health, and suffers from constipation, acne spots, or errors of refraction, such as hypermetropia or hypermetropic astigmatism. Until suppuration actually occurs, hot boric acid fomentations should be used, and the patient should be purged. When suppuration has occurred, the eyelash, which is usually in the centre of the yellow area where the pus is pointing, should be pulled out, and then, if necessary, the swelling should be incised, and again hot boric acid fomentations applied. Syrupus ferri phosphatic, in drachm doses, should be given twice or three times daily after food. Calcium sulphide, in doses varying from 1-8 to 1-2 a grain for an adult, given twice daily, has been recommended in cases of recurrent styes. When the more acute inflammatory symptoms have disappeared, the following ointment may be prescribed:

R Ungeunti hydrargyri oxidi flavi, .................... pt. j;
Petrolati, ....................................... pt. ij.
Ft. ung.

A small piece of the ointment to be applied to the margins of the eyelids with a fine camel hair brush night and morning. All errors of refraction must be corrected by the use of appropriate glasses. A generous diet, plenty of open air exercise, and, if possible, a change of air are also indicated.—The Practitioner, March, 1907.

